# The Stromal Vascular Fraction from Canine Adipose Tissue Contains Mesenchymal Stromal Cell Subpopulations That Show Time-Dependent Adhesion to Cell Culture Plastic Vessels

**DOI:** 10.3390/ani13071175

**Published:** 2023-03-27

**Authors:** Gabriele Scattini, Martina Pellegrini, Giulio Severi, Monica Cagiola, Luisa Pascucci

**Affiliations:** 1 Department of Veterinary Medicine, University of Perugia, 06123 Perugia, Italy; 2 Istituto Zooprofilattico Sperimentale dell’Umbria e delle Marche “Togo Rosati”, 06126 Perugia, Italy

**Keywords:** mesenchymal stromal/stem cells, dog, adipose tissue, stromal vascular fraction, immunophenotype, cell culture

## Abstract

**Simple Summary:**

Adipose tissue-derived mesenchymal stromal cells (MSCs) are stem-like cells extensively studied for regenerative applications in both human and veterinary medicine. Their isolation is usually performed by enzymatic digestion of adipose tissue followed by cell seeding. Tissue remnants that are still floating 48 h after seeding are discarded. We observed that waste tissue fragments still contain cells that adhere belatedly to the dish. In this study, we aimed to investigate their basic properties to speculate on the possible existence of MSC subpopulations. Samples of subcutaneous adipose tissue from three dogs were enzymatically digested. We obtained three cell populations that adhered to the culture dish 48, 96, and 144 h after seeding. Cells were analyzed through different methods to assess possible differences in phenotype, viability, proliferation, and differentiation ability. No significant differences were found between the three subpopulations. However, this procedure has proven to be a valuable method for dramatically improving MSC yield. As a consequence of cell recovery optimization, the amount of tissue harvested could be reduced, and the time required to obtain sufficient cells for clinical applications could be shortened. Nevertheless, functional differences cannot be excluded, and other assays are needed to investigate possible different biological properties.

**Abstract:**

Adipose-derived mesenchymal stromal cells (MSCs) are extensively studied in both human and veterinary medicine. Their isolation is usually performed by collagenase digestion followed by filtration and removal of nonadherent tissue remnants 48 h after seeding. We observed that waste tissue fragments contain cells that adhere belatedly to the plastic. We aimed to investigate their basic properties to speculate on the possible existence of MSC subpopulations. Adipose tissue from three dogs was enzymatically digested. Three cell populations that adhered to the culture plastic 48, 96, and 144 h after seeding were obtained. After expansion, they were analyzed by flow cytometry for MSC-positive (CD90, CD44, and CD29) and -negative (CD14, MHCII, and CD45) markers as well as for endothelial, pericyte, and smooth muscle cell markers (CD31, CD146, and alpha-SMA). Furthermore, cells were assessed for viability, doubling time, and trilineage differentiation ability. No significant differences were found between the three subpopulations. As a result, this procedure has proven to be a valuable method for dramatically improving MSCs yield. As a consequence of cell recovery optimization, the amount of tissue harvested could be reduced, and the time required to obtain sufficient cells for clinical applications could be shortened. Further studies are needed to uncover possible different functional properties.

## 1. Introduction

Mesenchymal stromal cells (MSCs) are a subset of heterogeneous fibroblastoid stromal cells with high self-renewal capacity and differentiation potential. In the last decades, interest in MSC has increased dramatically in human and veterinary medicine. This is confirmed by the numerous clinical trials aimed at validating their efficacy in a wide range of diseases (https://clinicaltrials.gov accessed on 1 March 2023), as well as the increasing number of reports on the clinical use of MSC in spontaneous animal diseases [1]. Due to their biological properties, MSC is currently the most extensively investigated cell type for advanced therapies. Their ability to modulate inflammatory/immunologic-related disorders, and their pro-regenerative potential, tropism for injured sites, and paracrine signaling make them suitable “smart” therapeutic tools [2]. Over time, MSCs have been obtained from many adult mammalian tissues and organs, including bone marrow, peripheral blood, synovial fluid, umbilical cord blood, Wharton jelly, placenta, spleen, and adipose tissue [3]. A position paper by the International Society for Cellular Therapy (ISCT) outlined the main basic characteristics of MSC [4]. According to the ISCT proposals, MSCs must have the ability to adhere to plastic when isolated from tissues and cultured in vitro; they must express several antigens such as CD90, CD73, and CD105 at a percentage ≥95%, and the typical hematopoietic surface molecules (CD45, CD34, and CD14) at a percentage ≤5%. Finally, they must be multipotent in vitro and display adipocytic, osteoblastic, and chondroblastic differentiation potential [4]. 

MSCs are increasingly used in dogs to treat a range of diseases, including orthopedic [5], digestive tract disorders [6,7], as well as diseases of the liver [8], kidney [9], heart [10], respiratory system, skin [11], eyes [12], and reproductive system. In addition, MSCs from dogs have been extensively studied as this species is of interest as a preclinical/clinical model for cell therapy in humans [13]. Various research groups have carried out the isolation of MSCs in dogs. Their characterization is usually performed by flow cytometry, immunofluorescence, or RT-PCR. Comparison between the different studies revealed some differences in ISCT indications for surface markers of MSC. The most common positive markers for canine MSCs are CD29, CD44, and CD90 [14,15,16,17]; of these, only CD90 is included in ISCT guidelines. CD73 has only been detected by flow cytometry in canine MSC by Russel et al. [16] and through RT-PCR in three published papers [14,15,18]. Expression of the marker CD105 by canine MSC has only been detected by [14] flow cytometry in two papers [19,20]. Various sources of MSCs are currently used in dogs. However, adipose tissue (AT) is one of the most studied as it is abundant, easy to obtain with minimally invasive procedures, and rich in MSCs that are relatively easy to isolate and propagate in vitro due to their high proliferative potential [21,22].

Isolation of MSCs from AT is usually performed according to a standardized procedure that includes the following stages: (1) mincing the tissue with scissors or a scalpel; (2) digestion by collagenase type I for 45 min to 1 h at 37 °C by gentle agitation in a water bath; (3) centrifugation and removal of floating lipid layer; (4) filtration of the stromal vascular fraction (SVF) through 100- and 70- or 40-µm filters; (5) washing and centrifugation; (6) removal of supernatant, resuspension of cell pellet, and seeding into a culture flask; (7) removal of the nonadherent cells from the culture 48 h after seeding. Comparing different protocols described in the relevant literature, it is clear that the main differences concern collagenase concentration and digestion time, but no other consistent differences emerge. The procedure described in most studies, therefore, represents a sequence of consolidated phases that are repeated fairly constantly in different laboratories [14,15,16,17,18,19,20,22].

In our many years of experience with the isolation and propagation of AT-derived MSCs from animal species, we have observed that vascular-stromal fragments obtained by enzymatic digestion of AT and typically eliminated by filtration or discarded at 48 h after seeding are capable of releasing cells over time that adhere to the plastic and proliferate for subsequent passages. Two possible hypotheses were formulated to explain this behavior: (i) The cells adhere at different time points but have the same biological identity. (ii) They adhere at different time points because they belong to different tissue niches and are biologically different. To test the correct hypothesis, we compared cells from three canine donors to highlight differences or similarities. For this purpose, three cell subpopulations were obtained based on their time-dependent affinity for plastic. They were compared in terms of viability, proliferative activity, immunophenotype, and differentiation ability towards adipogenic, osteogenic, and chondrogenic lineages, as suggested by ISCT.

## 2. Materials and Methods

### 2.1. Harvesting and Processing of Adipose Tissue

Five grams of subcutaneous adipose tissue were collected from three dogs under five years of age that had died of traumatic causes and were referred to the OVUD (University Veterinary Teaching Hospital) of the University of Perugia. The donors were female mixed-breed, medium-sized dogs. AT was collected from normal-weight dogs along the ventral midline of the abdomen. AT was collected a few hours after death sterilely, with the owners’ consent, who donated the cadavers for teaching and research purposes. The tissue samples were washed three times in phosphate-buffered saline (PBS) supplemented with 200 U/mL penicillin, 200 µg/mL streptomycin, and 250 ng/mL amphotericin B (Merck, Darmstadt, Germany). The tissue samples were finely minced with forceps and digested with 0.075% collagenase type I (Worthington Biochemical Corp., Lakewood, NJ, USA) at 37 °C for 75 min. The digested sample was centrifuged at 600 g for 10 min; the lipid fraction was discarded, while the pellet containing the SVF was seeded without filtration in two T25 tissue culture flasks (TPP, Trasadingen, Switzerland) and placed at 37 °C with 5% CO_2_ in DMEM low glucose (Dulbecco’s Modified Eagle Medium; Gibco, Gaithersburg, MD, USA) supplemented with 10% fetal bovine serum (FBS), 100 U/mL penicillin, and 100 µg/mL streptomycin for 48 h. The cells obtained from the first 48-h culture were named 1st Adhesion (Ad1).

The SVF residues floating in the medium were replated in two new T25 flasks (2nd adhesion—Ad2) to allow the adhesion of the remaining cells. After 48 h, the suspended tissue remnants were recovered and seeded again in two new T25 flasks (3rd adhesion—Ad3). At this stage, 20% of fresh medium was added to renew the exhausted components. The adherent cells of Ad1, Ad2, and Ad3 were maintained under the same culture conditions. When the cells reached 80% confluence, they were detached with trypsin-EDTA solution, counted by trypan blue dye exclusion, and seeded into new culture flasks with 2 × 10^4^ cells/cm^2^.

### 2.2. Proliferative Potential

The proliferative potential was calculated to estimate each population’s cumulative cell number and doubling time at P3. Cell populations Ad1, Ad2, and Ad3 were counted at each passage, from P1 to P4. The cumulative cell number (CN) was calculated using the following formula: CN = (nP1 × nP2 × nP3 × nP4)/(sP2 × sP3 × sP4); nP—number of cells counted after each passage; sP—number of cells seeded at each passage [23].

Doubling time was calculated for Ad1, Ad2, and Ad3 at P3. Cells were seeded in triplicate in a 48-well plate at 10^4^ cells/cm^2^. After four days, cells were detached and counted by trypan blue dye exclusion. Doubling time was calculated between day 1 and day 4 using the following formula: DT =72 h × [ln(2)/ln(n2/n1)]; n2—number of cells on day 4; n1—number of cells on day 1 [24].

### 2.3. Differentiation Potential

Ad1, Ad2, and Ad3 at P3 were seeded in a 24-well plate at 3 × 10^4^ cells/cm^2^ and incubated to confluence in DMEM supplemented with 10% FBS, 100 U/mL penicillin, and 100 µg/mL streptomycin.

For adipogenic differentiation, cells were treated with an induction medium (DMEM-LG, 10% FBS, 100 U/mL penicillin, 100 µg/mL streptomycin, 0.5 mM isobutyl methylxanthine, 100 µM indomethacin, 1 µM dexamethasone, and 10 µg/mL insulin) alternating with a maintenance medium (DMEM-LG, 10% FBS, 100 U/mL penicillin, 100 µg/mL streptomycin, and 10 µg/mL insulin). Adipogenesis induction lasted for 21 days, changing the medium every two days. At the end of the procedure, cells were labeled with the intravital fluorescent dye LipidSpot (Biotium, Fremont, CA, USA). The dye was diluted to a concentration of 1% in phenol red-free DMEM and applied to the culture for 30 min at 37 °C before being observed under a fluorescence microscope (Nikon Eclipse E800, Nikon Corporation, Tokyo, Japan) [25].

For osteogenic differentiation, cells were cultured in DMEM-LG supplemented with 10% FBS, 100 U/mL penicillin, 100 µg/mL streptomycin, 100 nM dexamethasone, 10 mM glycerophosphate, and 50 µM ascorbate-2-phosphate. Osteogenic induction lasted 21 days, with the medium changed every two days. At the end of the procedure, cells were fixed in 10% formalin and stained with Alizarin S [25].

For chondrogenic differentiation, cells were cultured in DMEM-LG supplemented with 1% FBS, 100 U/mL penicillin, 100 µg/mL streptomycin, 6.5 µg/mL insulin, 10 ng/mL TGF beta-3, and 50 nM ascorbate-2-phosphate. Induction lasted 21 days, and the medium was changed every two days. Chondrogenic differentiation was performed in microcentrifuge tubes to obtain micro-mass pellets. The samples were fixed with 10% formalin and embedded in paraffin. Five µm sections were stained with Alcian blue pH 1 [25].

### 2.4. Reverse Transcriptase PCR (RT-PCR)

Total RNA from differentiated cells was extracted using Trizol reagent and purified using PureLink RNA Mini Kit (Invitrogen) according to the manufacturer’s instructions. The isolated RNA was quantified using Nanodrop. cDNA was then obtained by reverse transcription of total RNA (500 ng per sample) using the High-Capacity cDNA RT Kit (Applied Biosystem - Thermo Fisher Scientific, Waltham, MA, USA) in a volume of 20 µl (thermal cycling condition: 25 °C for 10 min, 37 °C for 120 min, and 85 °C for 5 min). Ten ng of cDNA was used as the template for PCR amplification (35 cycles; denaturation at 95 °C for 15 s, annealing at temperatures indicated in Table 1 for 20 s, extension at 72 °C for 20 s). PCR products were separated on a 2% agarose gel in TAE (tris-acetate-EDTA) buffer.

Table 1 shows the sequences of the forward and reverse primers and the annealing temperature and product size for each gene.

### 2.5. Cell Viability

Ad1, Ad2, and Ad3 cell populations at P3 were seeded in a 96-well plate at a concentration of 3 × 10^4^ cells/cm^2^ and incubated for 48 h. Cell viability was determined using the Vybrant MTT assay (Invitrogen - Thermo Fisher Scientific, Waltham, MA, USA) according to the manufacturer’s instructions. Optical density was measured at 570 nm using the Tecan Infinite 200 microplate reader.

### 2.6. Immunophenotypic Analysis

A flow cytometric assay was performed to assess the expression of positive (CD90, CD29, and CD44) and negative (CD45, CD14, and MHC2) MSC markers commonly used in canine species. CD146, CD31, and alpha-SMA were also assessed to determine the presence of pericytes, endothelial cells, and smooth muscle cells in the three subpopulations.

At 80% confluence, Ad1, Ad2, and Ad3 cells at P3 were harvested from the culture flask, counted, and resuspended in incubation buffer (PBS-0.5% BSA) to 0.5 × 10^6^ cells/100 µL for each antibody tested. The primary antibodies used are listed in Table 2.

A permeabilization step was required to label alpha-actin. Briefly, 0.5 × 10^6^ cells were resuspended in 200 µL of fixation buffer and incubated for 10 min at room temperature. After washing at 400 g for 10 min with sterile phosphate-buffered saline (PBS, ph 7.4), the cells were resuspended in 100 µL of permeabilization buffer.

Antibodies were diluted in PBS according to the manufacturer’s instructions, added to the cell suspension for 15 min at room temperature in the dark, and stirred in a shaker. Finally, the cells were washed (400 g for 10 min) and resuspended in a fluorescence buffer. Data were recorded using FACS Calibur (Becton Dickinson, Franklin Lakes, NJ, USA) equipped with a laser BLUE 488 nm. Data analysis was performed using the CellQuest Pro (Becton Dickinson Immunocytometry Systems. Franklin Lakes, NJ, USA) and Kaluza 1.1 (Beckman Coulter, Brea CA, USA). Unstained cells were used as a control to detect autofluorescence.

## 3. Results

Modifications to the classical isolation protocol carried out in this study have shown that the vascular-stromal fragments, usually eliminated by filtration or discarded at 48 h post-seeding, contain cells capable of adhering to the culture plastic and proliferating for subsequent passages. The cell subpopulations Ad1, Ad2, and Ad3 had a fibroblastoid shape and showed no morphological differences in culture.

### 3.1. Proliferative Potential Min

The cumulative cell number was estimated for Ad1, Ad2, and Ad3 by counting cells after all passages and calculating the growth ratio between the number of cells seeded and those recovered after the growth period. The estimated cell number of the three populations at passage 4 was 5.20 ± 1.20 billion for Ad1, 6.39 ± 1.00 for Ad2, and 5.29 ± 1.42 for Ad3.

**Figure 1:**
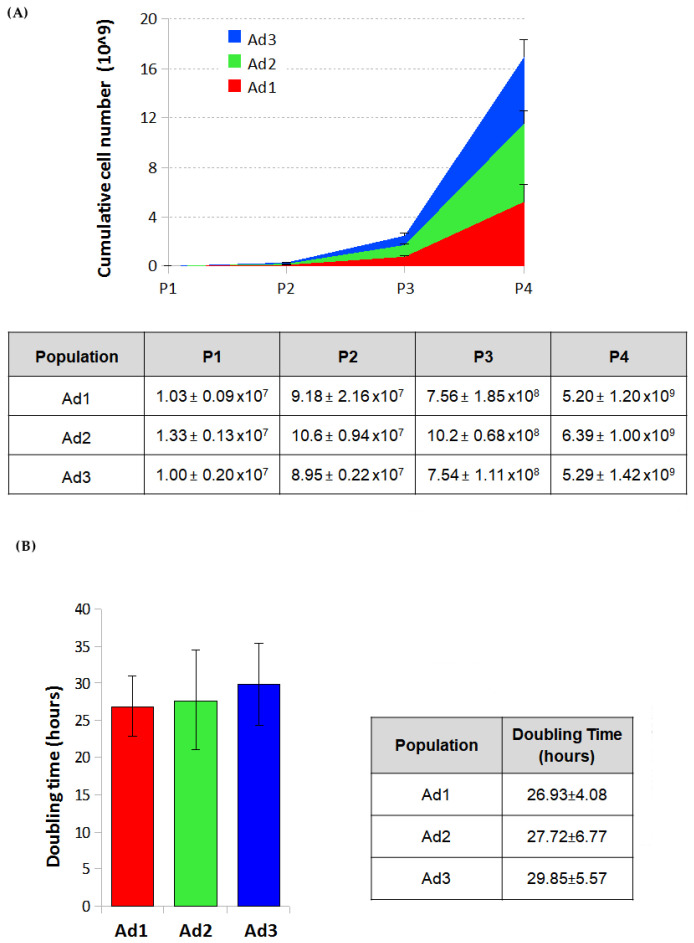
(**A**) Cumulative cell number of the three populations. Average number of cells obtained for the three populations (Ad1, Ad2, and Ad3) at different passages from five grams of dog adipose tissue. Mean values of the three dogs. (**B**) Doubling time of the three populations. Comparison of doubling of the three populations (Ad1, Ad2, and Ad3). Histograms represent the mean value calculated from three different dogs.

The proliferative capacity of Ad1, Ad2, and Ad3 at P3 was determined by calculating the doubling time over four days on P3 cells. The g-graphs obtained from the counting data show the trend of cell growth of the three subpopulations for four days. No significant differences were observed when cells from the same donor were considered. On the other hand, because of individual variability, minor differences were found among cells from different donors. The doubling time of the three populations ranged from 22 to 35 h. The mean doubling time was 26.93 ± 4.08 h for Ad1, 27.72 ± 6.77 for Ad2, and 29.85 ± 5.57 for Ad3 (Figure 1).

### 3.2. Differentiation Potential

**Figure 2:**
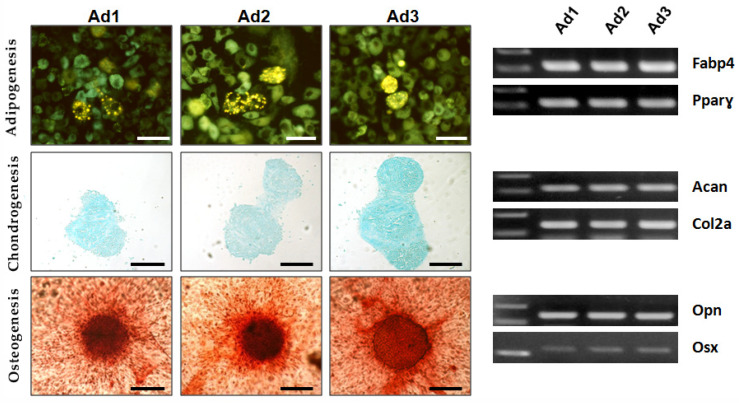
Multilineage differentiation. Representative images of trilineage differentiation induced on the 3 cell populations under study. White scale bars, 50 µm; Black scale bars, 200 µm. RT-PCR products for tissue-specific mRNA; FABP4 and PPARG2 for adipogenic differentiation; OPN and OSX for osteogenic differentiation; ACAN and COL2A1 for chondrogenic differentiation.

The differentiation potential of Ad1, Ad2, and Ad3 at P3 was examined by inducing the cells to differentiate into adipogenic, osteogenic, and chondrogenic lineages in vitro. Osteogenic and chondrogenic differentiation were confirmed by histochemical methods (alizarin S and alcian blue). In adipogenic differentiation, intracellular fluorescent dye lipid spots revealed the presence of multiple yellow–green fluorescent lipid droplets in the differentiated cells. RT-PCR confirmed the expression of tissue-specific genes in all three populations: osteopontin (OPN) and osterix (OSX) for osteogenic differentiation, fatty acid binding protein (FABP4), and peroxisome proliferator-activated receptor gamma (PPARγ) for adipogenic differentiation, aggrecan (ACAN), and collagen 2a1 (COL2A1) for chondrogenic differentiation. The results showed that all subjects’ Ad1, Ad2, and Ad3 were characterized by trilineage differentiation potential, as expected according to ISCT guidelines for MSCs (Figure 2).

### 3.3. Cell Viability

**Figure 3:**
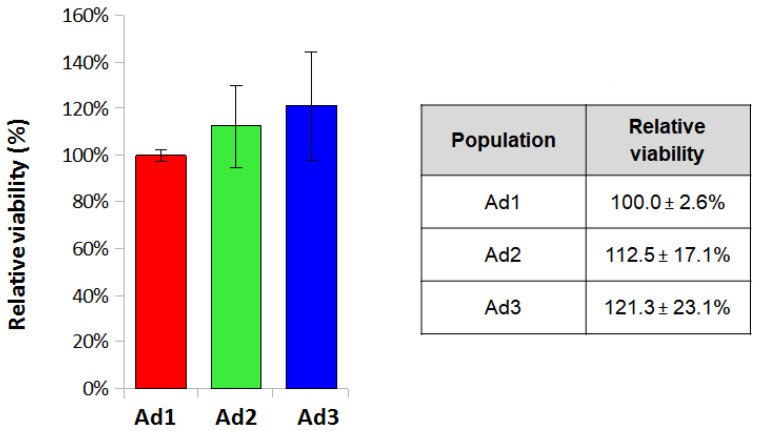
Relative viability of the three populations. Viability of the three populations (Ad1, Ad2, and Ad3) was measured with MTT assay. Absorbance values were normalized for the AD1 value. Histograms represent the mean relative viability values calculated for the three donor dogs.

The MTT assay was performed to assess cell viability by seeding Ad1, Ad2, and Ad3 cells at P3 at a density of 3.2 × 10^4^/cm^2^. Cell viability values obtained by MTT assay were normalized for the Ad1 population and analyzed for each donor to eliminate differences deriving from individual variability. The mean values for the three populations were 100 ± 2.6%, 112.5 ± 17.1%, and 121.3 ± 23.1%. The results showed that relative viability was comparable among the three populations since the values did not show statistically significant differences (Figure 3).

### 3.4. Immunophenotypic Analysis

The immunophenotypic profile of the three subpopulations was investigated by flow cytometry. The results were influenced by individual variability. Nevertheless, the cell populations did not differ in size and granularity, although cell aggregates corresponding to irregular fluorescence peaks were found. The high autofluorescence of the cells made an accurate assessment of weakly positive events complex and probably led to an underestimation of values and increased uncertainty.

**Figure 4:**
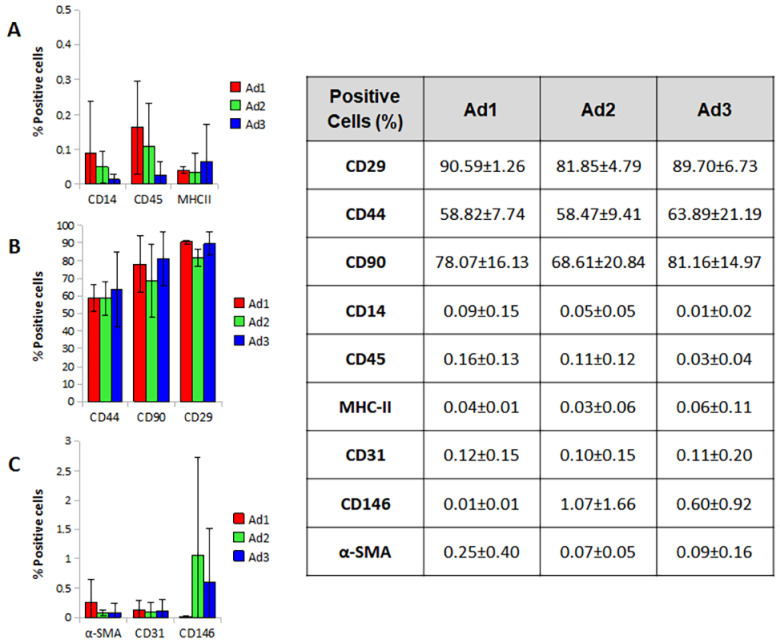
Immunophenotype profiles. Representative histograms of positive cells for MSC negative markers (**A**), positive markers (**B**), and other stromal markers (**C**). Mean values were calculated for the three dogs.

Ad1, Ad2, and Ad3 showed no differences in the expression of the markers tested; the only parameter that showed a statistically significant difference was CD29 in the Ad2 population, which showed a lower value compared to Ad1 (Ad2 81.85% vs. Ad1 90.59%) (*p*-value 0.038). Overall, the three populations were positive for CD29, CD44, and CD90, albeit with a value lower than the 95% parameter reported by ISCT. The three populations were negative for CD14, CD45, and MHCII, with values largely below the 2% threshold suggested by ISCT. Markers for pericytes, smooth muscle, and endothelial cells (CD146, a-SMA, and CD31) were not expressed or were weakly expressed by the cells studied (Figure 4).

## 4. Discussion

This research is based on the observation that SVF obtained by conventional enzymatic digestion of AT contains fibroblastoid cells that can adhere to the culture plastic at different time points after seeding. The study aimed to investigate the possible existence of subpopulations of AT-derived MSCs, characterized by different biological features that justify their delayed adherence. In particular, two hypotheses were formulated: (i) the cells that adhere at different time points have the same identity; (ii) the cells that adhere at different time points are biologically different. Both hypotheses have quite different consequences and may have significant implications in veterinary medicine, where—unlike in humans—MSCs are increasingly used in clinical practice. If the first hypothesis is confirmed, we will have developed a technical procedure that dramatically increases MSC yield and thus reduces the amount of sampled tissue and shorten the time needed between isolation and therapeutic application. This is one of the major drawbacks of MSC treatment, as prolonged in vitro expansion may be required to obtain a clinical ‘dose’ of cells. However, long-term culture may compromise the inherent properties of MSCs, resulting in the upregulation of senescent genes, morphological changes, reduced differentiation potential, and decreased immunomodulatory properties [26]. The procedure for isolating and expanding MSCs described in this study made it possible to overcome some of these issues by significantly increasing cell yield. Our data show that it is possible to obtain up to 2.5 billion MSCs at P3 from five grams of AT, or 17 billion at P4, considering the sum of the three subpopulations.

On the contrary, if the second hypothesis is confirmed, we will have demonstrated the presence of different tissue niches harboring cells with different biological properties. This evidence would have significant implications both in vitro and in vivo, and it would require further thorough investigation, as different biological characteristics can be associated with different therapeutic potentials.

To test which hypothesis is correct, we characterized the three cell subpopulations based on the minimum requirements defined by the ISCT for MSCs. Specifically, we examined their trilineage multipotency and molecular phenotype. We also compared their proliferative potential by estimating doubling time and viability. The three cell subsets identified in this study overlapped in their proliferation potential, as doubling time and MTT assays revealed no significant differences.

Together with plastic adherence and immunophenotype, multipotency assessment is a common and reliable method for identifying MSCs. In this study, the three subgroups were comparable in their ability to undergo trilineage differentiation, as demonstrated by histochemical staining and RT-PCR.

Concerning immunophenotype, the most commonly used molecular pattern to identify MSCs was confirmed in all three subgroups. However, the data reported in the relevant literature on the phenotype of MSCs in dogs are somewhat inconsistent with regard to the quantitative expression of surface markers. Indeed, CD29, CD90, and CD44 were found to be reliable positive markers in dogs, with percentages of expression not exceeding 95% (as suggested by the ISCT for human MSC markers) but consistent with previous studies [14,15,16] and not differing significantly among the three subpopulations. Similarly, negative markers were always less than 2%. Evaluation of markers related to stromal-vascular cell populations distinct from MSCs (pericytes, smooth muscle cells, and endothelial cells) showed that these cells were always present in a minimal percentage (<5%).

In sum, considering the ISCT suggestions and the related species specificities, the features highlighted in Ad1, Ad2, and Ad3 lead them all to be identified with MSCs. It can be assumed that tissue fragments that remain in suspension release MSCs over time that are sequestered inside the minced tissue and not reached by collagenase. It could be assumed that the MSC niche is maintained in the suspended fragments. It can also be hypothesized that after stimulation by factors present in the medium or released by cells already adherent to the plastic, the residual MSCs entrapped in the SVF proliferate and increase in number before being released, justifying the high cell yield associated with this procedure. Although these conclusions are suggestive, they are not supported by confirmatory data at the moment and can only be speculative.

The authors also believe that the problem of the identity of MSCs cannot be exhaustively solved using only the minimum parameters established by ISCT. For example, it cannot be excluded that there is a hierarchy of cells in AT that share the same basic characteristics but are distinguished by specific markers not considered in this study and are associated with different tissue niches. In a paper by Merrick et al., the authors showed that there are subpopulations of stromal cells with immunophenotypic features located in different tissue areas. The most immature cells are in the so-called reticular stroma, a compartment located around adipocyte aggregates or specific organs [27]. Using these unconventional markers and in situ examination of resident MSCs would provide additional elements to clarify their location and density in canine adipose tissue.

Furthermore, potency assays could provide interesting additional data to understand whether the biological identity demonstrated by ISCT parameters also corresponds to a functional identity. In this context, it should be emphasized that the biology and function of MSCs depend on a variety of mechanisms and on soluble and insoluble messages that, despite the phenotypic similarity between cells, can be very different and can produce dramatically different effects in vitro and in vivo.

## 5. Conclusions

In conclusion, we herein demonstrated that AT-SVF could release over time much more MSCs than those usually recovered with the classical procedure. In vitro analyses confirmed their identity based on the parameters suggested by ISCT. This study may seem purely methodological; indeed, in light of the results, we have only developed a new technique of isolation and large-scale expansion of MSCs, which allows a high cell yield. If we consider the rapid development of cell therapies in the veterinary field and the need for large quantities of cells to be administered, this is a significant result as it produces an undoubted advantage for developing clinical applications of MSCs.

However, if we go beyond the methodological aspect, this study raises new crucial questions about the distinctive features of MSCs and suggests a revision of the criteria used for their identification. Indeed, characterizing MSCs based on nonspecific phenotypic markers or on their differentiating ability is not exhaustive and completely ignores the possibility of identifying subclasses of MSCs with different biological properties. In order to develop more targeted therapeutic approaches, moving to a functional assessment of the identity of MSCs by potency assays could allow dissecting of their biological properties, significantly enhancing their clinical potential.

## Figures and Tables

**Table 1:** List of primers for tissue-specific mRNA in dogs.

Gene	Accession Number	Forward Primer Sequence	Reverse Primer Sequence	Annealing Temperature	Product Size
OPN	XM_038444089	AGAGAAGTGCAGCATCGTCC	CACAGCATTCTGCTTTTCCTCA	60 °C	170 bp
OSX	XM_038438062	ACGACACTGGGCAAAGCAG	CATGTCCAGGGAGGTGTAGAC	60 °C	285 bp
FABP4	XM_038441472	ATCAGTGTAAACGGGGATGTG	GACTTTTCTGTCATCCGCAGTA	57 °C	117 bp
PPARɣ	NM_001024632	ACACGATGCTGGCGTCCTTGATG	TGGCTCCATGAAGTCACCAAAGG	63 °C	119 bp
ACAN	NM_001113455	ATCAACAGTGCTTACCAAGACA	ATAACCTCACAGCGATAGATCC	56 °C	122 bp
COL2A1	NM_001006951	GAAACTCTGCCACCCTGAATG	GCTCCACCAGTTCTTCTTGG	56 °C	157 bp
GAPDH	NM_001003142	TGTCCCCACCCCCAATGTATC	CTCCGATGCCTGCTTCACTACCTT	60 °C	100 bp
B2M	XM_038441716	TCTACATTGGGCACTGTGTCAC	TGAAGAGTTCAGGTCTGACCAAG	60 °C	136 bp

ACAN (aggrecan), COL2A1 (collagen 2 type 1a), FABP4 (fatty acid binding protein), OPN (osteopontin), OSX (osterix, transcription factor Sp7), and PPARɣ (peroxisome proliferator-activated receptor gamma). Housekeeping genes: GAPDH (Glyceraldehyde-3-Phosphate Dehydrogenase) and B2M (beta-2-microglobulin).

**Table 2:** List of labeled antibodies for canine cell characterization.

Antibody	Manufacturer	Catalog Number
Alpha-Smooth Muscle Actin Antibody (SPM332) PE	Novusbio (Centennial, CO, USA)	NBP2-34760PE
CD14 Monoclonal Antibody (Tuk4) FITC	ThermoFisher Scientific (Carlsbad, CA, USA)	MA1-82074
CD29 anti-human Antibody (TS2/16) PE	BioLegend (San Diego, CA. USA)	303004
CD90 Monoclonal Antibody (YKIX337.217) PE	ThermoFisher Scientific (Carlsbad, CA, USA)	12-5900-42
CD44 Monoclonal Antibody (YKIX337.8) FITC	ThermoFisher Scientific (Carlsbad, CA, USA)	11-5440-42
CD45 Monoclonal Antibody (YKIX716.13) PE	Biorad (Hercules, CA, USA)	MCA1042PE
CD146 Monoclonal Antibody (P1H12) FITC	ThermoFisher Scientific (Carlsbad, CA, USA)	11-1469-42
CD31 Polyclonal Antibody PE	Bioss Antibodies (Woburn, MA, USA)	bs-0468R-PE
Rat anti dog MHC ClassII monomorphic (YKIX334.2) FITC	Biorad (Hercules, CA, USA)	MCA1044F

## Data Availability

No additional data are available.
